# Charing Cross Hospital

**Published:** 1910-11-19

**Authors:** Juliet Duff

**Affiliations:** President of the Appeal Committee.


### EDITOR'S LETTER-BOX.

### CHARING CROSS HOSPITAL.

LETTER FROM LADY JULIET DUFF.—APPEAL FOR £100,000.

SIR,—As President of the Appeal Committee formed to raise the sum of £100,000 to free Charing Cross Hospital from all debt and enable the wards at present closed to be reopened, I am asking for space in your valuable columns to appeal to the public for their generous support.

The urgent need of the Charing Cross Hospital arises from the fact that it has a large mortgage debt, and the annual payments for interest and sinking fund in respect of this debt are a tremendous drain upon its income. In consequence the Council have been forced to close several wards in order to reduce expenditure. Five wards, containing eighty-seven beds, are now closed, which means that some 1,500 sick persons are deprived of the benefits of this charity every year. These closed wards and b[illegible] could all be filled, for the waiting-list of the hospital has always from eighty to a hundred names upon it, and the less serious of these cases have to wait for weeks, and sometimes months, before being admitted. Even casualties that ought to be taken in have frequently to be sent on to other hospitals, or to the infirmary, because the beds are full.

This is one of the most serious aspects of the present insufficient accommodation, for it must be remembered that Charing Cross Hospital, from its very situation, is pre-eminently an accident hospital. Cases are brought in from all parts of the busy neighbourhood around—from business houses, hotels, clubs, restaurants, factories, railway stations, and from the streets themselves, so increasingly dangerous since the advent of motor traffic. Moreover, the adjacent thoroughfares are highways for all sorts and conditions of people from all over the world, and the hospital is called upon to perform not merely a local but a universal service.

Its record of unwearying devotion to the alleviation of sickness and suffering extends back for seventy-six years, and its present position of indebtedness is due to the fact in the course of time the buildings had become totally inadequate and out of date, and to make the hospital efficient a large scheme of reconstruction was necessary. Those who have known the old hospital will realise that the increased efficiency was worth the price, but unhappily, till the price is paid, the sick and suffering are defrauded of a large part of the benefit that should be theirs.

I therefore appeal to the wealthy and generous-hearted to pay the price by redeeming the debt, so that the hospital may enter upon the full service which is now denied to it.

Donations sent to me either at Charing Cross Hospital or at 51 Portland Place, W., will be most gratefully acknowledged.—Yours faithfully,

JULIET DUFF,
President of the Appeal Committee.

